# Characterization of glomerular basement membrane components within pediatric glomerular diseases

**DOI:** 10.1093/ckj/sfae037

**Published:** 2024-02-14

**Authors:** Dan Chen, Xindi Zhou, Chun Gan, Qing Yang, Wanbing Chen, Xiaoqian Feng, Tao Zhang, Li Zhang, Lujun Dai, Yaxi Chen, Haiping Yang, Mo Wang, Wei Jiang, Qiu Li

**Affiliations:** Department of Nephrology, Children's Hospital of Chongqing Medical University, National Clinical Research Center for Child Health and Disorders, Ministry of Education Key Laboratory of Child Development and Disorders, Chongqing Key Laboratory of Pediatric Metabolism and Inflammatory Diseases, Chongqing, P.R. China; Department of Nephrology, Children's Hospital of Chongqing Medical University, National Clinical Research Center for Child Health and Disorders, Ministry of Education Key Laboratory of Child Development and Disorders, Chongqing Key Laboratory of Pediatric Metabolism and Inflammatory Diseases, Chongqing, P.R. China; Department of Nephrology, Children's Hospital of Chongqing Medical University, National Clinical Research Center for Child Health and Disorders, Ministry of Education Key Laboratory of Child Development and Disorders, Chongqing Key Laboratory of Pediatric Metabolism and Inflammatory Diseases, Chongqing, P.R. China; Department of Nephrology, Children's Hospital of Chongqing Medical University, National Clinical Research Center for Child Health and Disorders, Ministry of Education Key Laboratory of Child Development and Disorders, Chongqing Key Laboratory of Pediatric Metabolism and Inflammatory Diseases, Chongqing, P.R. China; Department of Nephrology, Children's Hospital of Chongqing Medical University, National Clinical Research Center for Child Health and Disorders, Ministry of Education Key Laboratory of Child Development and Disorders, Chongqing Key Laboratory of Pediatric Metabolism and Inflammatory Diseases, Chongqing, P.R. China; Department of Nephrology, Children's Hospital of Chongqing Medical University, National Clinical Research Center for Child Health and Disorders, Ministry of Education Key Laboratory of Child Development and Disorders, Chongqing Key Laboratory of Pediatric Metabolism and Inflammatory Diseases, Chongqing, P.R. China; Chongqing University Three Gorges Hospital, School of Medicine of Chongqing University, Chongqing, P.R. China; Pediatric Renal Immunology Specialist Section, The Affiliated Hospital of Guizhou Medical University, Guizhou Provincial Children's Medical Center, Guiyang, Guizhou, P.R. China; Pediatric Renal Immunology Specialist Section, The Affiliated Hospital of Guizhou Medical University, Guizhou Provincial Children's Medical Center, Guiyang, Guizhou, P.R. China; Department of Pathology, The Affiliated Hospital of Guizhou Medical University, Guiyang, Guizhou, P.R. China; Centre for Lipid Research & Key Laboratory of Molecular Biology for Infectious Diseases (Ministry of Education), Institute for Viral Hepatitis, Department of Infectious Diseases, the Second Affiliated Hospital, Chongqing Medical University, Chongqing, P.R. China; Department of Nephrology, Children's Hospital of Chongqing Medical University, National Clinical Research Center for Child Health and Disorders, Ministry of Education Key Laboratory of Child Development and Disorders, Chongqing Key Laboratory of Pediatric Metabolism and Inflammatory Diseases, Chongqing, P.R. China; Department of Nephrology, Children's Hospital of Chongqing Medical University, National Clinical Research Center for Child Health and Disorders, Ministry of Education Key Laboratory of Child Development and Disorders, Chongqing Key Laboratory of Pediatric Metabolism and Inflammatory Diseases, Chongqing, P.R. China; Department of Nephrology, Children's Hospital of Chongqing Medical University, National Clinical Research Center for Child Health and Disorders, Ministry of Education Key Laboratory of Child Development and Disorders, Chongqing Key Laboratory of Pediatric Metabolism and Inflammatory Diseases, Chongqing, P.R. China; Department of Nephrology, Children's Hospital of Chongqing Medical University, National Clinical Research Center for Child Health and Disorders, Ministry of Education Key Laboratory of Child Development and Disorders, Chongqing Key Laboratory of Pediatric Metabolism and Inflammatory Diseases, Chongqing, P.R. China

**Keywords:** collagen IV α3α4α5, glomerular basement membrane, glomerular disease, integrin α3β1, laminin α5β2γ1

## Abstract

**Background:**

Disruptions in gene expression associated with the glomerular basement membrane (GBM) could precipitate glomerular dysfunction. Nevertheless, a comprehensive understanding of the characterization of GBM components within pediatric glomerular diseases and their potential association with glomerular function necessitates further systematic investigation.

**Methods:**

We conducted a systematic analysis focusing on the pathological transformations and molecular attributes of key constituents within the GBM, specifically Collagen IV α3α4α5, Laminin α5β2γ1, and Integrin α3β1, across prevalent pediatric glomerular diseases.

**Results:**

We observed upregulation of linear expression levels of COL4A3/4/5 and Laminin 5α proteins, along with a partial reduction in the linear structural expression of Podocin in idiopathic nephrotic syndrome (INS), encompassing minimal change disease (MCD) and focal segmental glomerulosclerosis (FSGS), but showing a reduction in IgA nephropathy (IgAN), IgA vasculitis nephritis (IgAVN) and lupus nephritis (LN). Furthermore, our study revealed reductions in Laminin β2γ1 and Integrin α3β1 in both primary and secondary childhood glomerular diseases.

**Conclusion:**

In INS, notably MCD and FSGS, there is a notable increase in the linear expression levels of COL4A3/4/5 and Laminin 5α proteins. In contrast, in IgAN, IgAVN, and LN, there is a consistent reduction in the expression of these markers. Furthermore, the persistent reduction of Laminin β2γ1 and Integrin α3β1 in both primary and secondary childhood glomerular diseases suggests a shared characteristic of structural alterations within the GBM across these conditions.

## Graphical Abstract

**Graphical Abstract:**
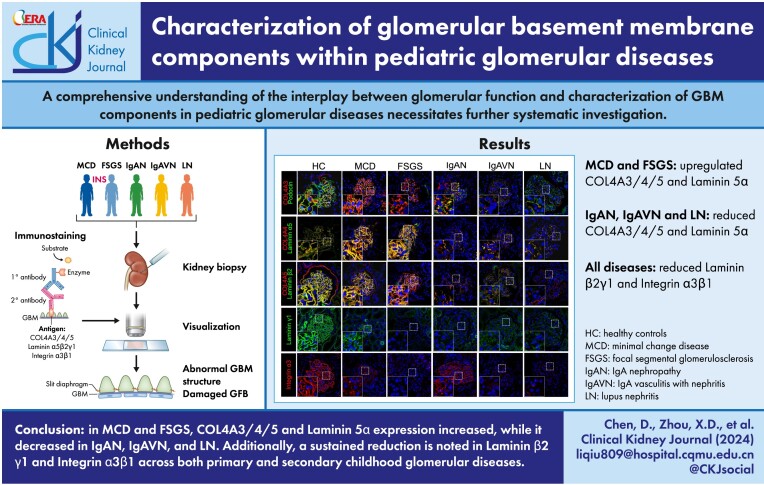

KEY LEARNING POINTS **What was known**:

- The glomerular basement membrane (GBM) is crucial for structural support and filtration in the kidney. Disturbances in GBM-related gene expression could lead to glomerular dysfunction.

**This study adds**:

- Our comprehensive study explored the molecular characteristics of key GBM components in pediatric glomerular diseases, revealing upregulation of COL4A3/4/5 and Laminin 5α in idiopathic nephrotic syndrome (INS), especially minimal change disease (MCD) and focal segmental glomerulosclerosis (FSGS), and a reduction in IgA nephropathy (IgAN), Immunoglobulin A vasculitis nephritis (IgAVN), and lupus nephritis (LN). Furthermore, Laminin β2γ1 and Integrin α3β1 were consistently reduced in primary and secondary pediatric glomerular diseases.

**Potential impact**:

- These findings illuminate the diverse molecular profiles in pediatric glomerular diseases and highlight the importance of understanding these variations for precise diagnosis and tailored treatments.

## INTRODUCTION

Glomerular diseases represent a frequent cause of kidney issues in pediatric patients, characterized by a spectrum of clinical features such as hematuria, proteinuria, and edema. Globally, these conditions account for 5% to 14% of chronic kidney disease (CKD) cases and 15% to 29% of end-stage renal disease (ESRD) cases among children [1, 2]. Notably, idiopathic nephrotic syndrome (INS) is the most prevalent among pediatric glomerular

diseases, responsible for 50% of cases and serving as the leading indication for kidney biopsies in children. Following closely is IgA nephropathy (IgAN) at 17%. In the pediatric population, IgA vasculitis nephritis (IgAVN) and lupus nephritis (LN) are the most common secondary glomerular diseases, comprising 13% and 9% of cases, respectively [3]. Remarkably, despite their distinct nature, these glomerular diseases are associated with structural alterations in the glomerular basement membrane (GBM).

The GBM, comprising condensed regions of extracellular matrix (ECM), is a pivotal component of the glomerular capillary wall, playing a crucial role in kidney filtration. It consists of essential components such as Collagen Type IV, specifically, Collagen IV α3α4α5 chains (encoded by *COL4A3/4/5*) interacting with the NC1 domain to form a trimer to provide structural support; Laminin, particularly the α5β2γ1 (encoded by *LAMA5, LAMB2, LAMC1*) isoform crucial for GBM stability; and Integrins, specifically Integrin α3β1 (encoded by *ITGA3, ITGB1*), are transmembrane receptors that play a vital role in connecting the GBM to the underlying podocytes and the glomerular endothelium, collectively forming a critical filtration barrier within the kidney's glomerulus, enabling selective substance passage while maintaining structural integrity [4–8]. The clinical manifestation of structural abnormalities in the GBM resulting from mutations in the *COL4A3, COL4A4*, and *COL4A5* genes is more pronounced in individuals with Alport syndrome (AS) and thin basement membrane nephropathy (TBMN) [9, 10]. Additionally, variants in *LAMB2* can lead to Pierson syndrome [11]. Children carrying homozygous deletion or missense mutations in *ITGA3* exhibit kidney features such as atrophic glomeruli, FSGS, diffuse interstitial fibrosis, tubular atrophy, proteinuria, as well as tubular loss and immaturity [12]. However, when excluding kidney diseases caused by genetic mutations, it is currently unknown how the expression patterns of these molecules are in kidney glomerular diseases and what relationship they have with changes in glomerular function.

INS, primarily attributed to minimal change disease (MCD) or FSGS, is characterized by abnormally high GBM permeability, leading to excessive excretion of proteins in the urine [13, 14]. Emerging evidence emphasizes the critical role of the glomerular filtration barrier (GFB) in causing proteinuria, with structural and functional changes in the GBM playing a central role in various kidney glomerular diseases [15, 16]. For instance, in IgA nephropathy (IgAN), which predominantly affects children, there is a characteristic deposition of IgA in the glomerular mesangium, leading to GBM changes [17–19]. Another notable glomerular disorder, IgA vasculitis, is a systemic condition that affects multiple organs, including the kidneys, and is frequently encountered in children [20, 21]. It is worth noting that structural and functional anomalies in the GBM may contribute to the pathogenesis of both IgAN and IgAVN, although the precise mechanisms are still undefined [20, 22]. Furthermore, alongside these aforementioned conditions, systemic lupus erythematosus (SLE), an autoimmune disease, often causes immune-related inflammation and damage to the GBM, particularly affecting the kidneys through LN [23, 24]. However, the significance of molecular changes in the GBM and the underlying processes contributing to the progressive decline in renal function in pediatric glomerular diseases are still not well understood.

Exploring molecular changes in the GBM associated with pediatric glomerular diseases is a crucial aspect of pediatric nephrology. It could unravel intricate disease mechanisms, guide tailored treatments, and hold promise in early disease detection, ultimately enhancing patient outcomes. Our comprehensive analysis of common pediatric glomerular diseases identified abnormal expressions of key GBM components, including Collagen IV α3α4α5, Laminin α5β2γ1, and Integrin α3β1, linked to GBM disruptions in primary and secondary glomerular conditions. These findings underscore the potential of these molecules as diagnostic and therapeutic targets, propelling the field of precision medicine and revolutionizing the diagnosis and treatment of these intricate conditions.

## MATERIALS AND METHODS

### Exome sequencing

Genomic DNA was extracted from whole blood using the QIAamp DNA Mini Kit (180134, Qiagen). A targeted approach was employed for gene capture, focusing on 97 genes associated with kidney disease. This was achieved through the utilization of the GenCap custom enrichment kit from MyGenostics Inc, following the recommended manufacturer's protocol. Subsequently, the enriched libraries underwent sequencing on an Illumina HiSeq 2000 sequencer (Illumina) for paired-end reads of 150 base pairs. The obtained reads were then aligned to the reference human genome (hg19) using the Short Oligonucleotide Analysis Package (SOAP) aligner software (SOAP2.21; soap.genomics.org.cn/soapsnp.html). Following alignment, single nucleotide polymorphisms (SNPs) were annotated using the SOAPsnp program, while deletions and insertions (InDels) were identified through the Genome Analysis Toolkit software 3.7. To ensure data quality, low-quality variations were filtered out based on a quality score of ≥20 and a minor allele frequency (MAF) of ≤0.01.

### Inclusion criteria and clinical data set

#### INS

The diagnosis of INS with biopsy-proven MCD or FSGS was established based on specific clinical indicators. These included the presence of edema, a 24-hour urinary protein excretion of ≥50 mg/kg, a morning urinary protein/creatinine ratio exceeding 2 mg/g, hypoalbuminemia characterized by serum albumin levels below 25 g/L, and a disease origin that remained unknown. Inclusion criteria for this group encompassed patients, whether they were newly diagnosed or experiencing a relapse of the underlying disease for more than three months, and were under the age of 18. Exclusion criteria comprised patients with non-nephrotic proteinuria, congenital nephrotic syndrome, and nephrotic syndrome secondary to metabolic, infectious, vascular, malignant, or cardiac conditions.

#### IgAN

Our study included cases of biopsy-confirmed IgAN in individuals under the age of 18. These cases met specific criteria, including an initial estimated glomerular filtration rate (eGFR) of ≥30 ml/min per 1.73 m^2^, initial proteinuria levels of ≥150 mg per 24 hours, and a minimum of 10 glomeruli available for analysis. Only cases with a follow-up period of at least 12 months, regardless of the duration of follow-up, were included in our analysis. We excluded cases with secondary causes of mesangial IgA deposits, such as IgAVN, and cases with comorbid conditions like diabetes mellitus.

#### IgAVN

All individuals between the ages of 1 and 18 years at the onset of their disease, who had received a clinical diagnosis of IgA vasculitis as per the criteria established by the American College of Rheumatology, and who also had a confirmed diagnosis of IgA vasculitis nephritis supported by renal biopsy displaying histologically predominant mesangial IgA immune deposits, were considered for inclusion in this study. Additionally, a minimum follow-up period of 6 months was required for eligibility [25].

#### LN

Renal involvement in patients under the age of 18 with SLE is characterized by specific criteria, including the presence of one or more of the following: a 24-hour urinary protein excretion of ≥150 mg (or a urine protein-to-creatinine ratio ≥0.2) or the presence of red blood cell casts in the urine. An additional valuable criterion for diagnosis is a renal biopsy revealing immune-complex-mediated nephritis, accompanied by complement deposition and varying degrees of cellular damage. It is imperative to order a renal biopsy whenever LN is suspected. For a standalone diagnosis of LN, patients must exhibit renal biopsy findings consistent with LN, coupled with elevated levels of antinuclear antibodies (ANA) and/or increased circulating levels of anti-double-stranded DNA (anti-dsDNA) antibodies.

This study included a total of 25 patients diagnosed with various glomerular diseases, including MCD, FSGS, IgAN, IgAVN, and LN. Information such as age, gender, and serum biochemical and urinary protein indexes were collected for each patient. The study protocol was approved by the Ethics Committee of the Children's Hospital of Chongqing Medical University under Approval No. (2022) Ethics Review (Research) No. 477 and conducted in accordance with the principles outlined in the Helsinki Declaration. Informed consent was obtained from all participants.

### Histological analysis and staining

The sample for light microscopy was fixed in neutral buffered formalin and embedded in paraffin using standard procedures. We stained paraffin sections with H&E, PAS, IHC, and IF, and captured digital images using an Olympus light microscope.

### Transmission electron microscopy (TEM)

We analysed TEM images using Image Pro plus 6.0 software. For each patient, we randomly selected four glomeruli and captured ten electron micrographs in each glomerulus.

### Immunohistochemistry

For immunohistochemical staining, paraffin sections were first deparaffinized and then treated with antigen retrieval buffer. Subsequently, they were blocked and incubated with primary antibodies, including anti-Integrin β1 (dilution of 1:50, #34971S, Cell Signaling Technology). The diaminobenzidine (DAB, DA1010, Solarbio) was used for color development. Digital images were captured using a light microscope (Olympus) with either a ×20 objective lens (low-power) or ×40 objective lens (high-power). ImageJ software was used for image analysis.

### Confocal and fluorescence microscopy

Kidney biopsies were fixed in neutral buffered formalin and embedded in either paraffin using standard procedures. We performed immunofluorescence staining on paraffin sections, and obtained images using a Nikon A1R Meta confocal microscope. For each sample, an average of six glomerular images was randomly selected. To ensure consistency, all images were captured under uniform parameters. Subsequently, the raw image files underwent analysis for average fluorescence intensity in local glomeruli using NIS-Element software. This meticulous approach was employed to maintain objectivity and rigor in the quantification process.

The antibodies used were list below: anti-Nephrin antibody (1:200, GP-N2, Progen), anti-Podocin antibody (1:200, PA5-79757, Invitrogen), anti-COL4A3 antibody (1:50, #7076, Chondrex), anti-LAMA5 antibody (1:200, ab210957, Abcam), anti-COL4A4 antibody (1:50, #7073, Chondrex), anti-COL4A5 antibody (1:50, ab231957, Abcam), anti-LAMA5 antibody (1:200, ab210957, Abcam), anti-LAMB2 antibody (1:200, ab210956, Abcam), anti-LAMC1 antibody (1:50, sc-17751, Santa Cruz), anti-ITGA3 antibody (1:50, Cat No.66070–1-Ig, Proteintech), goat anti-Guinea Pig IgG (H + L) highly cross-adsorbed secondary antibody Alexa fluor 647 (1:400, A-21235, Invitrogen), goat anti-Mouse IgG (H + L) cross-adsorbed secondary antibody Alexa fluor 568 (1:500, A-11075, Invitrogen), goat polyclonal secondary antibody to rabbit Alexa fluor 488 (1:400, ab150077, Abcam), goat polyclonal secondary antibody to rat Alexa fluor 647 (1:400, ab150159, Abcam), goat polyclonal secondary antibody to mouse Alexa fluor 488 (1:400, ab150113, Abcam), DAPI (1:1000, C1002, Beyotime).

### Statistical analysis

All data were analysed using GraphPad Prism 9 (Macintosh). Quantitative values are presented as the mean ± s.d. Statistical differences between two experimental groups were analysed by 2-tailed Student's *t*-test. For multiple comparison analysis, one-way ANOVA followed by Tukey's multiple comparison tests was performed.

## RESULTS

### Exploring GBM abnormalities and molecular insights in children with INS

While the identification of structural changes in podocytes associated with INS has been largely achieved, the significance of glomerular function and the pathological changes in the GBM remain poorly understood. To address these gaps, we investigated GBM structures and lesions in children diagnosed with INS, specifically those with MCD and FSGS. Notably, the clinical presentation of these individuals aligns with the diagnostic criteria for INS, which encompassed the presentation of edema, a 24-hour urinary protein excretion of ≥50 mg/kg, a morning urinary protein/creatinine ratio exceeding 2 mg/mg, hypoalbuminemia indicated by serum albumin levels falling below 25 g/L, and the presence of an undisclosed underlying cause for the disease. Additionally, it is noteworthy that these patients did not manifest mutations in genes associated with the GBM, as identified through exome sequencing (ES). Histopathological examination, involving hematoxylin-eosin (H&E), periodic acid-silver methenamine (PASM), Masson and periodic-acid Schiff (PAS) staining, revealed marked distinctions between children with MCD and FSGS in comparison to the healthy control (HC) group. FSGS exhibited more extensive mesangial expansion, pronounced GBM thickening, and segmental sclerosis (Fig. S1A and B, see online supplementary material). Additionally, FSGS displayed more severe interstitial fibrosis and a substantial reduction in podocyte foot processes, underscoring the impact of abnormal GBM on GFB function in children with INS (Fig. S1A and B, see online supplementary material). In summary, these findings suggest that the abnormal GBM in children with INS may impact GFB function, underscoring the importance of investigating GBM composition in the diagnosis and treatment of INS.

**Figure 1:**
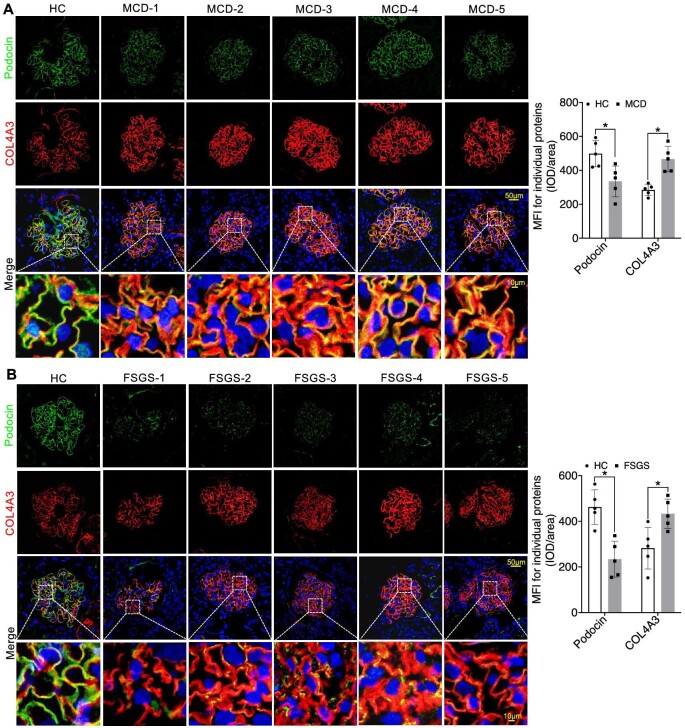
Significant linear increased in COL4A3 expression accompanied by varying degrees of Podocin expression decrease in MCD and FSGS compared to the HC Group (**A** and **B**). IOD, integrated optical density; HC, health control. *P < 0.05.

**Figure 2:**
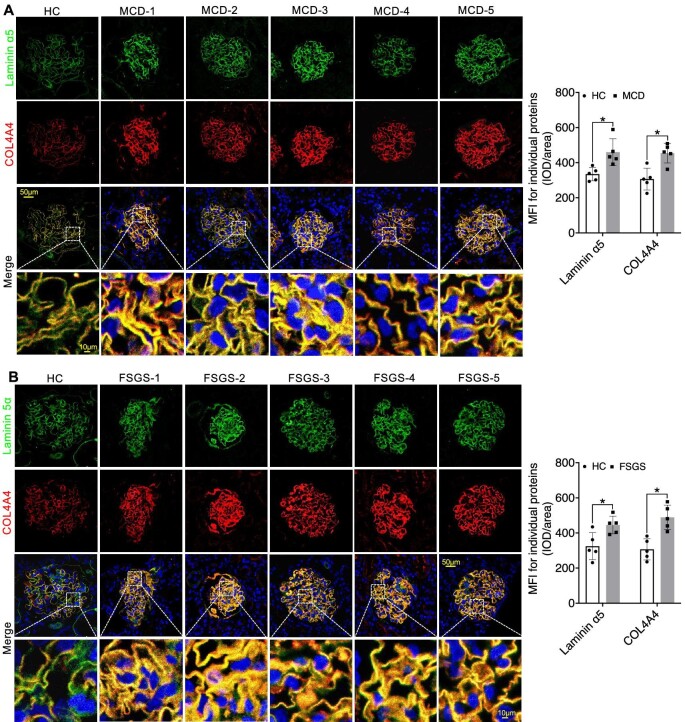
IF staining revealed elevated expression of COL4A4 and Laminin α5 in MCD and FSGS compared to the HC Group (**A** and **B**). *P < 0.05.

**Figure 3:**
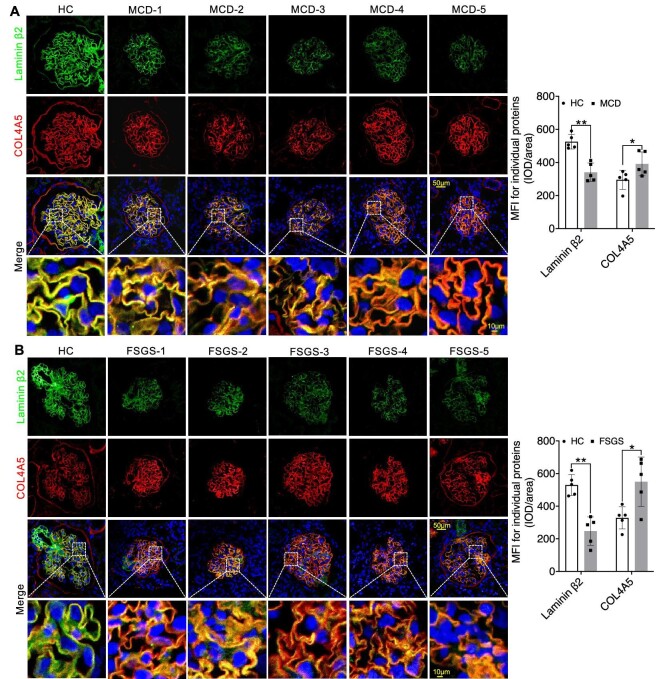
IF staining revealed varying degrees of reduction in Laminin β2 expression in MCD and FSGS compared to the HC Group, with a significant increase in COL4A5 expression in FSGS (**A** and **B**). ns, not significant. *P < 0.05; **P < 0.01.

**Figure 4:**
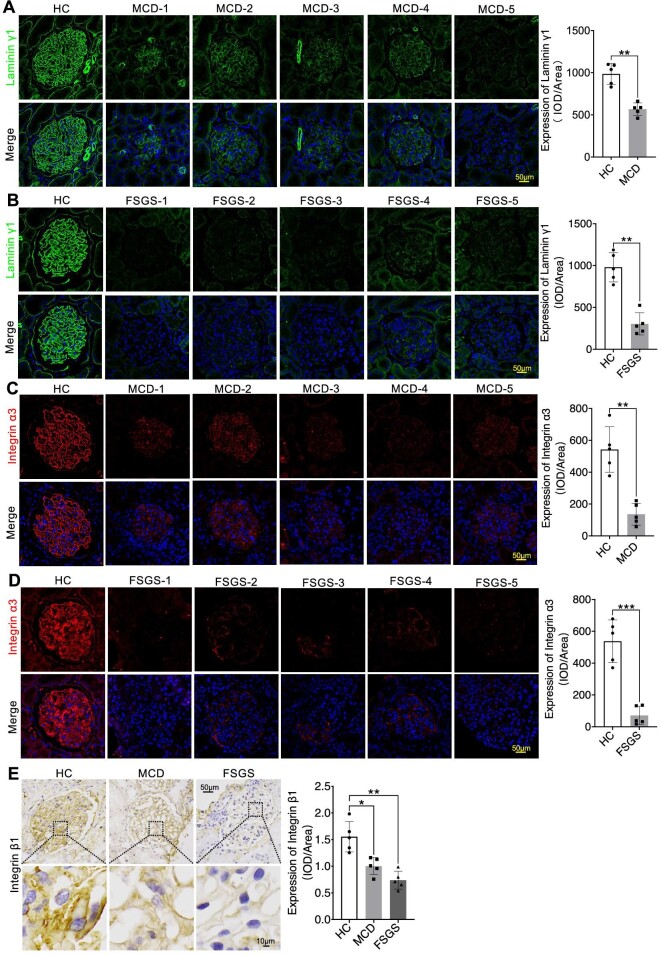
Immunostaining indicated the significantly decreased expression of Laminin γ1 and Integrin α3β1 in MCD and FSGS compared to the HC Group (**A**–**E**). *P < 0.05; **P < 0.01; ***P < 0.001.

In our quest to thoroughly investigate the precise subcellular localization and structural changes of multiple molecules within the GFB, we employed multi-channel immunofluorescence (IF) to analyse the expression patterns of various molecules in kidney biopsy samples. Our primary objective was to establish a connection between the expression of key components of the GBM in glomeruli and the functioning of podocytes in INS. We initially concentrated on these pivotal proteins, including Collagen IV α3α4α5, Laminin α5β2γ1, and Integrin α3β1, along with slit diaphragm-related proteins Nephrin and Podocin. We observed a significant linear increase in COL4A3/4/5 expression as well as the mesangial cell marker PDGFRβ in both MCD and FSGS cases when compared to HC group, accompanied by structural alterations in Podocin and Nephrin, resulting in a decrease in their expression levels (Figs 1A and B, 2A and B, 3A and B; Fig. S1C and D, see online supplementary material). Nevertheless, the expression of Laminin displayed inconsistencies. Specifically, Laminin 5α exhibited elevated expression in both MCD and FSGS cases, while Laminin β2 and laminin γ1 demonstrated decreased expression (Figs 2A and B, 3A and B, 4A and B). Additionally, the expression of Integrin proteins, including Integrin α3 and Integrin β1, was diminished in MCD and FSGS (Fig. 4C–E). Collectively, these findings suggested that a more severe aberrant expression of type IV collagen α3α4α5, Laminin α5β2γ1, and Integrin α3β1 may be associated with renal dysfunction, as indicated by serum creatinine concentration, blood urea nitrogen, proteinuria, and eGFR (Table S1, see online supplementary material), suggesting a possible association with derangements within the GBM of INS.

### Exploring expression patterns of GBM components in IgAN

Having established the expression patterns of the crucial components of the GBM, including Collagen IV α3α4α5, Laminin α5β2γ1, and Integrin α3β1, in individuals with INS, our next goal was to investigate whether comparable patterns could be identified in individuals with IgAN. To achieve this, we conducted a comprehensive assessment of renal morphological changes and clinical characteristics in patients diagnosed with IgAN, ensuring that they met the predefined inclusion criteria for our study. We employed a range of staining techniques, including H&E, PASM, Masson, and PAS, to visualize and analyse different tissue structures and glycoproteins. These techniques confirmed the presence of mesangial hyperplasia with increased mesangial cells in the glomeruli, uneven GBM, interstitial fibrosis, and segmental sclerosis, which are consistent with the diagnostic criteria for IgAN (Fig. S2A, see online supplementary material).

**Figure 5:**
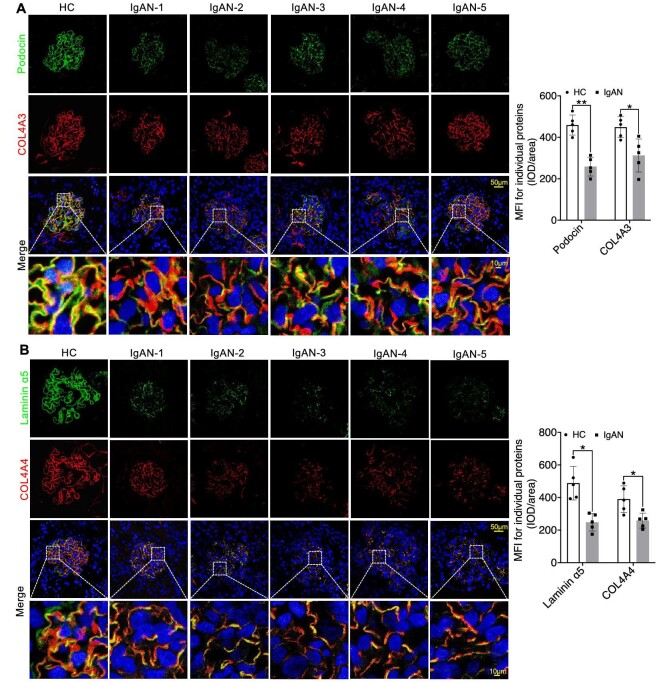
Significant linear decrease in COL4A3/4 and Laminin α5 expression, along with a significant decrease in Podocin expression in IgAN compared to the HC Group (**A** and **B**). *P < 0.05; **P < 0.01.

**Figure 6:**
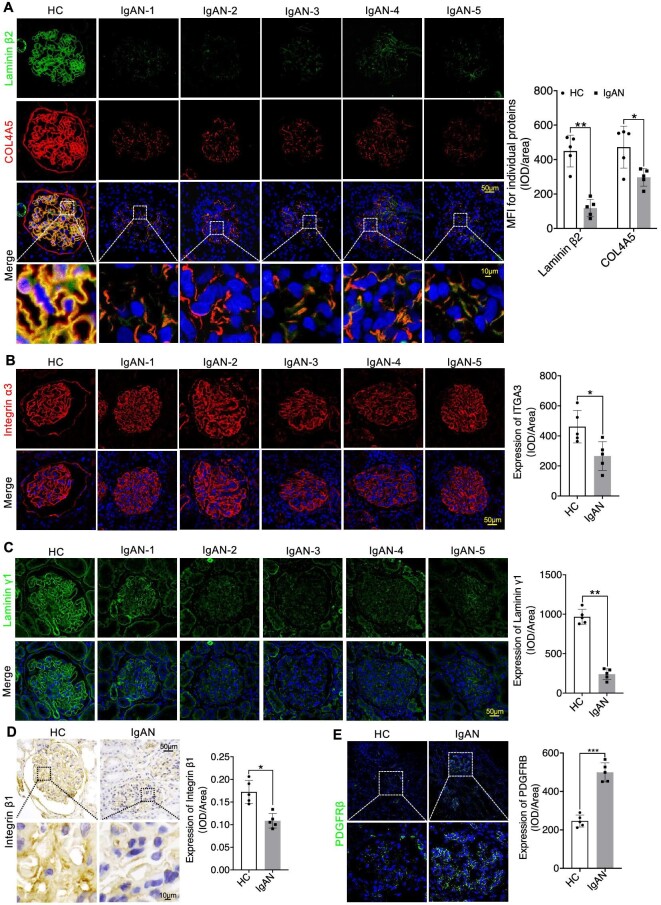
Immunostaining indicated the significantly decreased expression of COL4A5 and Laminin β2 (**A**), Integrin α3 (**B**), Laminin γ1 (**C**) and Integrin β1(**D**), but increased expression of PDGFRβ (**E**) in IgAN compared to the HC Group. ns, not significant. *P < 0.05; **P < 0.01; ***P < 0.001.

We further employed IF and confirmed the expression of COL4A3/4/5 in IgAN. In contrast to INS, we observed substantial disruption to the linear structure of COL4A3/4/5, as well as Podocin, which was coupled with a reduction in their expression levels (Figs 5A and B, and 6A). Additionally, the linear structure of Laminin α5β2γ1 and Integrin α3β1 was also markedly compromised, leading to a decrease in its expression (Figs 5B and 6A–D). Concurrently, there was an observed increase in the expression of PDGFRβ (Fig. 6E). In aggregate, these findings indicated the presence of aberrant expressions of type IV Collagen α3α4α5, Laminin α5β2γ1, and Integrin β1α3 in IgAN. These anomalies engendered changes in the thickness of the basement membrane, prompting podocytes to respond by elongating their foot processes. Ultimately, these adaptations imposed structural constraints that culminate in podocyte impairment and renal dysfunction, further evidenced by abnormalities in biochemical indicators of renal function (Table S1, see online supplementary material).

### Expression patterns of the key GBM proteins in IgAVN and LN

Based on the aforementioned results, it was conceivable that the expression profiles of Collagen IV α3α4α5, Laminin α5β2γ1, and Integrin α3β1 may exhibit variations in IgAVN and LN. To achieve a comprehensive understanding of these protein profiles in the context of IgAVN and LN development, further investigations were imperative. Consequently, we conducted a study to assess the expression patterns of these proteins in patients with IgAVN and LN, encompassing individuals with diverse clinical characteristics who met the inclusion criteria outlined in the Materials and Methods section. Furthermore, the histopathological analyses, encompassing H&E, PASM, Masson, and PAS staining, provided additional confirmation of the presence of renal morphological changes consistent with the diagnostic criteria for IgAVN and LN (Fig. S2B, see online supplementary material).

**Figure 7:**
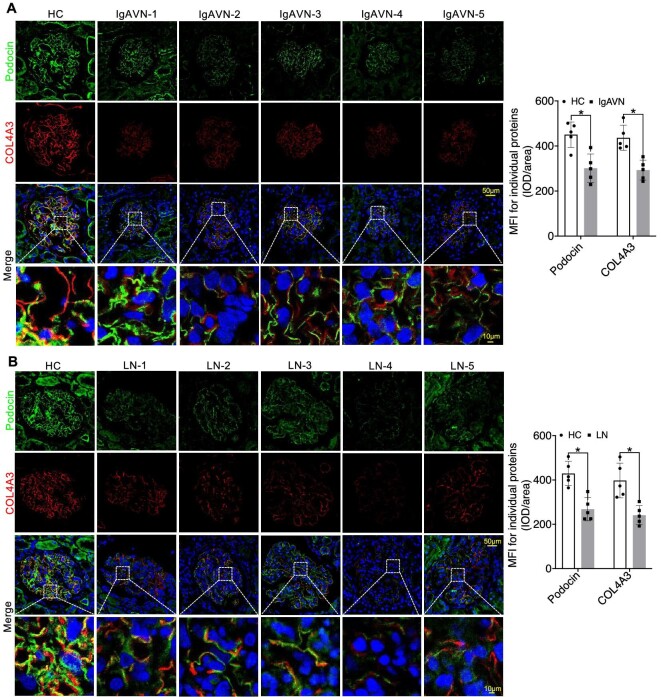
IF staining revealed significant reduction in Podocin and COL4A3 expression in IgAVN and LN compared to the HC Group (**A** and **B**). *P < 0.05.

**Figure 8:**
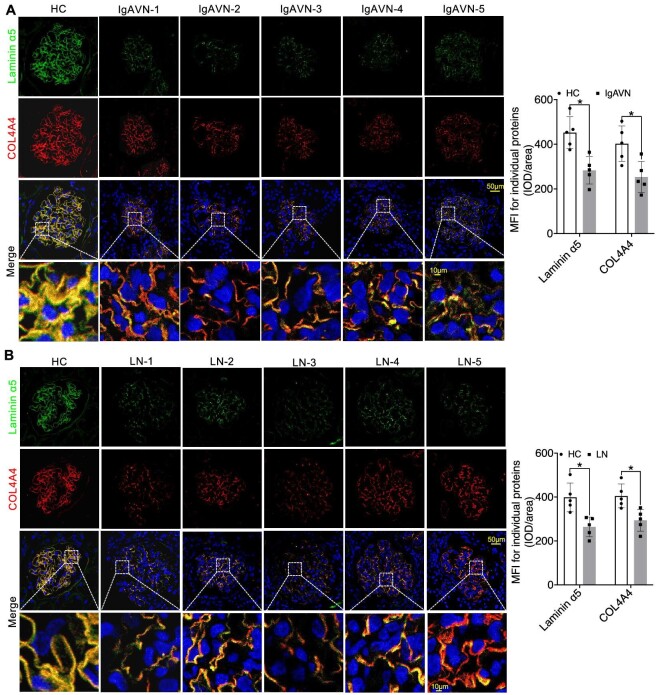
IF staining indicated the significantly decreased expression of Laminin α5 and COL4A4 in IgAVN and LN compared to the HC Group (**A** and **B**). *P < 0.05.

**Figure 9:**
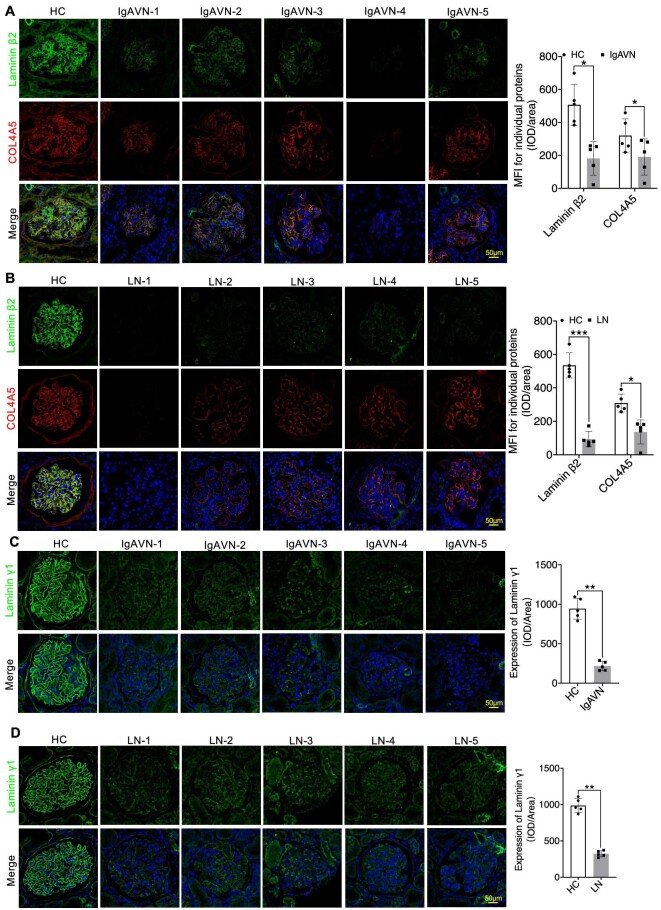
IF staining revealed varying degrees of reduction in Laminin β2γ1 and COL4A5 expression in IgAVN and LN compared to the HC Group (**A**–**D**). *P < 0.05; **P < 0.01; ***P < 0.001.

**Figure 10:**
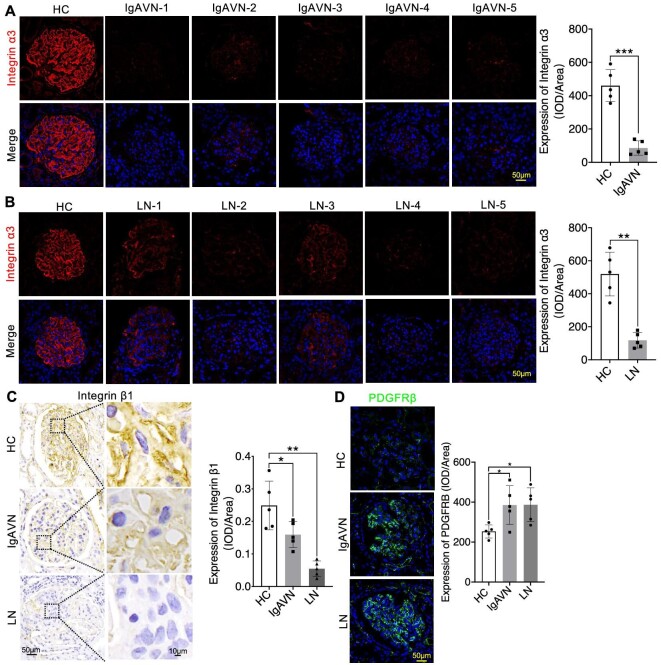
Immunostaining revealed significantly varying degrees of reduction in Integrin α3 and Integrin β1 expression but increased expression of PDGFRβ in IgAVN and LN compared to the HC Group (**A**–**D**). *P < 0.05; **P < 0.01; ***P < 0.001.

In our investigation into the expression patterns of Collagen IV α3α4α5, Laminin α5β2γ1, and Integrin α3β1 in IgAVN and LN, we conducted additional IF analyses. Our findings consistently revealed a significant reduction in COL4A3/4/5 expression, as well as Podocin. This reduction was prominently characterized by the narrowing of the linear protein expression structure (Figs 7A and B, 8A and B, and 9A and B). Similarly, Laminin α5β2γ1 consistently exhibited a uniform expression pattern across all analysed samples, and Integrin α3β1 displayed a similar expression pattern, except for PDGFRβ (Figs 8A and B, 9A–D, and 10A–D). These findings strongly suggest the presence of aberrant expression of Collagen IV α3α4α5, Laminin α5β2γ1, and Integrin α3β1 in both IgAVN and LN, and the degree of abnormal expression may have implications for biochemical indicators related to renal function (Table S1, see online supplementary material).

## DISCUSSION

The GBM serves as a critical barrier in the GFB physiology, preventing the passage of molecules from the bloodstream into urine [6, 26]. Disruptions in the GBM structure and thickness due to aberrant expression of specific molecular markers like COL4A3/4/5, Laminin α5β2γ1 and Integrin α3β1 can lead to GFB dysfunction and, consequently, glomerular diseases. Thus, comprehending changes in GBM composition is essential for accurately diagnosing and precisely treating glomerular diseases in children. However, a notable gap exists in systematic research regarding GBM composition profiles, underscoring the urgent need for further investigation into molecular markers and alterations associated with various pediatric glomerular diseases and individual patients. This research is crucial for ensuring accurate diagnoses and effective treatment strategies.

The kidneys and the immune system share a profound interconnection, with renal autoantigens frequently becoming targets of pathogenic immune responses. Notably, systemic autoimmunity, in contrast to idiopathic podocytopathies like MCD and FSGS, can lead to localized manifestations involving the formation of abnormally proliferating immune complexes that target mesangial cells. This phenomenon is exemplified in conditions such as IgAN, IgAVN, and LN, as evidenced by the significantly increased expression of PDGFRβ. In the initial stages of immune-mediated glomerular inflammation, the interaction between immune complexes and specific Fc receptors on both infiltrating leukocytes and resident mesangial cells assumes a pivotal role. This interaction triggers the activation of the complement system and sets in motion local inflammatory processes. Subsequently, activated mesangial cells release substantial amounts of inflammatory mediators, precipitating extensive damage, including harm to endothelial cells and podocytes [27–29]. Importantly, podocytes, responsible for producing components of the glomerular basement membrane, become a source of structural disorders within the membrane under these inflammatory conditions.

In this study, we systematically delineated the characterization of GBM components within pediatric glomerular diseases and their potential association with glomerular function. COL4A3/4/5 and Laminin α5β2γ1 were identified as pivotal factors influencing GBM thickness, while Integrin α3β1 was found to be perturbed in both types of glomerular diseases. Specifically, in the context of INS with MCD and FSGS, our findings revealed an upregulation in the linear expression levels of COL4A3/4/5 proteins, accompanied by a partial reduction in the linear structural expression of Podocin. However, in contrast to COL4A3/4/5, the expression pattern of Laminin α5β2γ1 did not demonstrate consistent trends. To provide more specific details, Laminin α5 exhibited increased expression, whereas Laminin β2γ1 displayed reduced expression, aligning with the expression pattern observed for Integrin α3β1. Furthermore, in IgA nephropathy, IgAVN, and LN, we obtained consistent findings where the linear structures of COL4A3/4/5, Laminin α5β2γ1, and Integrin α3β1 were all significantly impacted, resulting in reduced protein expression. In summary, we established links between abnormal marker expression and GBM disruptions in both primary and secondary glomerular diseases, highlighting the pivotal roles of COL4A3/4/5 and Laminin α5β2γ1 in influencing GBM thickness, alongside perturbations in Integrin α3β1. These findings demonstrated consistency across various glomerular diseases, emphasizing a substantial impact on the linear structural expression of proteins.

Collagen type IV, forming three triple helix trimers through the NC1 domain (including the α3α4α5 network predominant in the mature GBM and distinct from the α1α1α2 basement membranes) is a vital structural protein within basement membranes [6]. The NC1 domain, situated at the C-terminal end of each alpha chain, plays a pivotal role in the trimerization of collagen type IV. Interactions within the NC1 domains are responsible for stabilizing these trimers, typically consisting of three alpha chains, where each chain contributes an NC1 domain that interacts with those of the other two chains [30]. These interactions involve various structural features, including disulfide bonds and non-covalent interactions, maintaining the integrity of the trimeric structure [31, 32]. The abnormal expression of α3α4α5, as observed in our study, may be linked to disruptions in the NC1 domain, potentially leading to trimer destabilization. However, further investigation is needed to understand the specific regulation of its spatial structure.

Laminins serve as the principal non-collagenous constituents of basement membranes and are composed of heterotrimeric structures comprising α, β, and γ chains. In the mature GBM, the predominant isoform is α5β2γ1 [6]. Notably, globular laminin (LG) domains are situated at the C-terminus of the α chain of laminin, and they form connections with the α-subunit of Integrin receptors found on cell surfaces. In podocytes, the primary receptor is Integrin α3β1, which directly interacts with laminin α5 through LG domains, facilitating essential cellular processes [6, 33, 34]. Our study has revealed that the abnormal expression of Laminin α5β2γ1 and Integrin α3β1, observed in primary and secondary pediatric glomerular diseases, leads to disruptions in GBM structure and thickness, potentially playing a significant role in proteinuria resulting from podocyte injury. These findings emphasize the importance of GBM composition in accurate diagnosis and targeted treatment of glomerular diseases in children.

The GBM is distinguished by dense regions of ECM, serving as a crucial separator between podocytes and endothelial cells. However, studying the basement membrane proves challenging in basic *in vitro* cell experiments, primarily due to the difficulty in effectively simulating the linear structure of the ECM. Recent advancements in organoid research offer promising avenues, providing potential insights into the intricate nature of the GBM and its functions [35, 36]. Examining GBM molecule expression in pediatric glomerular diseases holds immense clinical and research significance, offering enhanced diagnostic precision and tailored care. It enables precise identification of disease types and severity, empowering healthcare professionals to provide accurate diagnoses, predict disease progression and treatment response, and may reveal novel therapeutic targets, potentially revolutionizing precision medicine. Investigating these molecular alterations in pediatric populations is crucial due to distinct presentations and responses, contributing to both patient care and the advancement of pediatric nephrology.

In summary, the GBM is integral to kidney filtration, and changes in its composition can lead to pediatric glomerular diseases. Our study investigated the abnormal expression of key molecules in the GBM, uncovering distinct patterns. Specifically, INS, including MCD and FSGS, displayed upregulated COL4A3/4/5 and Laminin 5α but a partial Podocin reduction, while IgAN, IgAVN, and LN showed reduced expression. Additionally, Laminin β2γ1 and Integrin α3β1 consistently decreased in primary and secondary pediatric glomerular diseases, potentially contributing to GBM disruptions and disease onset. Maintaining molecular balance in the GBM is crucial (see the Graphical Abstract). This research enhances our understanding of pediatric GBM and kidney disease mechanisms.

## Supplementary Material

sfae037_Supplemental_Files

## Data Availability

All data from this study are available upon request by contacting the corresponding author.
