# Supplementary material for: Characterization of glomerular basement membrane components within pediatric glomerular diseases
Source: Clin Kidney J. 2024 Feb 14;17(3):sfae037. doi: 10.1093/ckj/sfae037 (PMC10919337; doi:10.1093/ckj/sfae037)
Supplement: sfae037_Supplemental_Files [file sfae037_supplemental_files.zip › Supplemental Table.pptx]

## Slide 1
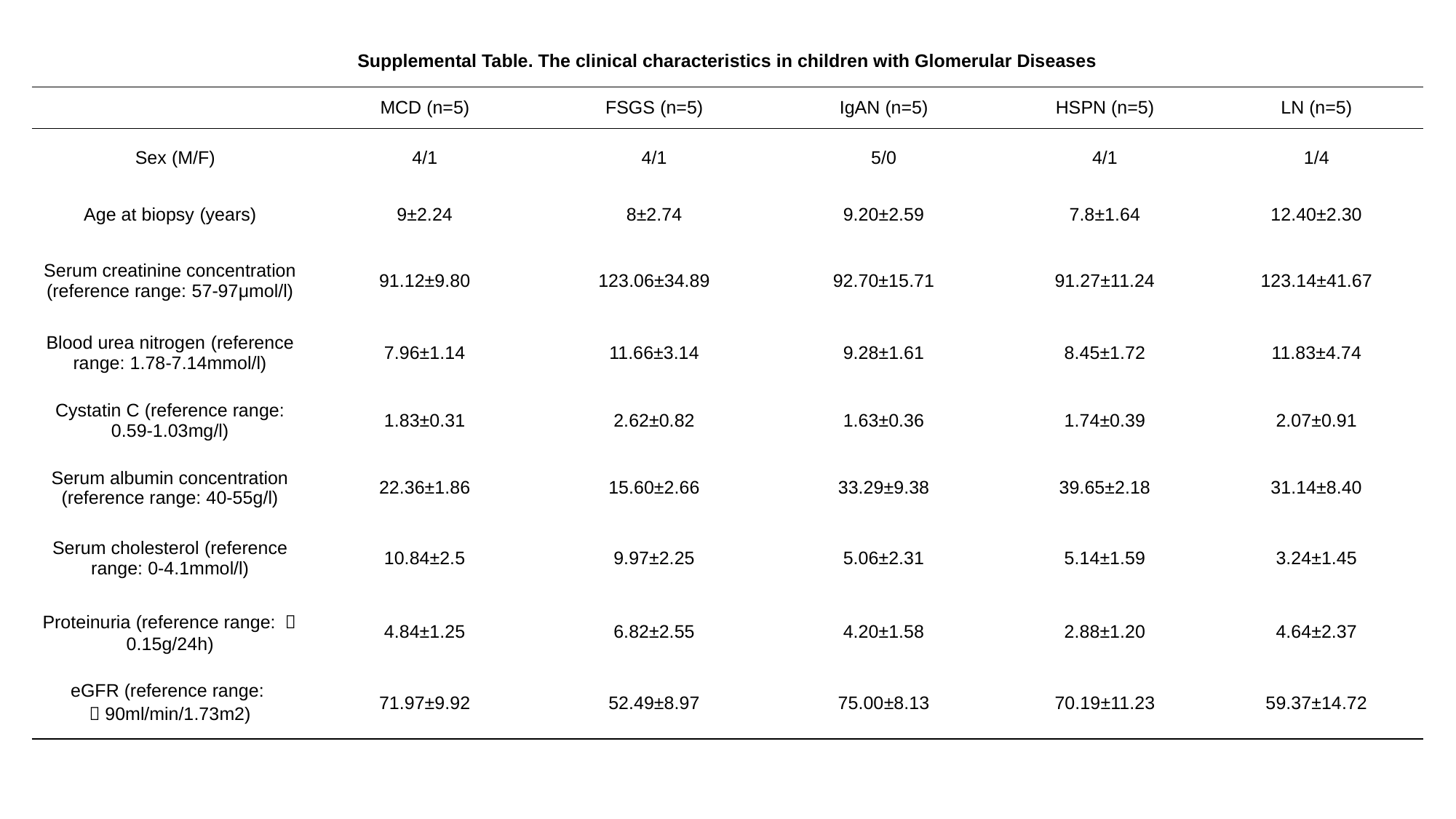

Supplemental Table. The clinical characteristics in children with Glomerular Diseases
| | MCD (n=5) | FSGS (n=5) | IgAN (n=5) | HSPN (n=5) | LN (n=5) |
| --- | --- | --- | --- | --- | --- |
| Sex (M/F) | 4/1 | 4/1 | 5/0 | 4/1 | 1/4 |
| Age at biopsy (years) | 9±2.24 | 8±2.74 | 9.20±2.59 | 7.8±1.64 | 12.40±2.30 |
| Serum creatinine concentration (reference range: 57-97μmol/l) | 91.12±9.80 | 123.06±34.89 | 92.70±15.71 | 91.27±11.24 | 123.14±41.67 |
| Blood urea nitrogen (reference range: 1.78-7.14mmol/l) | 7.96±1.14 | 11.66±3.14 | 9.28±1.61 | 8.45±1.72 | 11.83±4.74 |
| Cystatin C (reference range: 0.59-1.03mg/l) | 1.83±0.31 | 2.62±0.82 | 1.63±0.36 | 1.74±0.39 | 2.07±0.91 |
| Serum albumin concentration (reference range: 40-55g/l) | 22.36±1.86 | 15.60±2.66 | 33.29±9.38 | 39.65±2.18 | 31.14±8.40 |
| Serum cholesterol (reference range: 0-4.1mmol/l) | 10.84±2.5 | 9.97±2.25 | 5.06±2.31 | 5.14±1.59 | 3.24±1.45 |
| Proteinuria (reference range: ＜0.15g/24h) | 4.84±1.25 | 6.82±2.55 | 4.20±1.58 | 2.88±1.20 | 4.64±2.37 |
| eGFR (reference range: ＞90ml/min/1.73m2) | 71.97±9.92 | 52.49±8.97 | 75.00±8.13 | 70.19±11.23 | 59.37±14.72 |
