# Supplementary figures and images for: Characterization of glomerular basement membrane components within pediatric glomerular diseases
Source: Clin Kidney J. 2024 Feb 14;17(3):sfae037. doi: 10.1093/ckj/sfae037 (PMC10919337; doi:10.1093/ckj/sfae037)

# Supplemental Figure 1

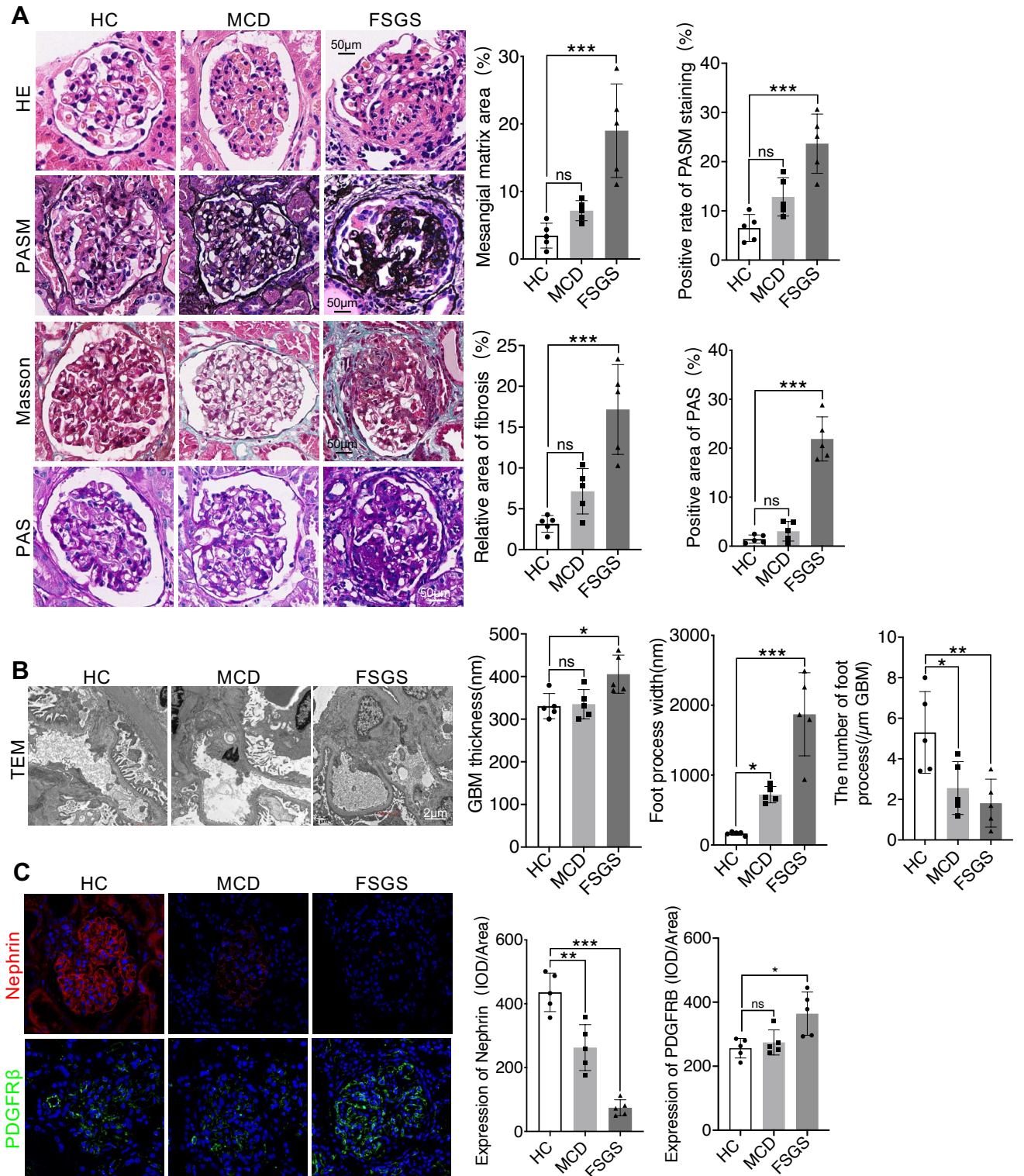

Supplemental Figure 2

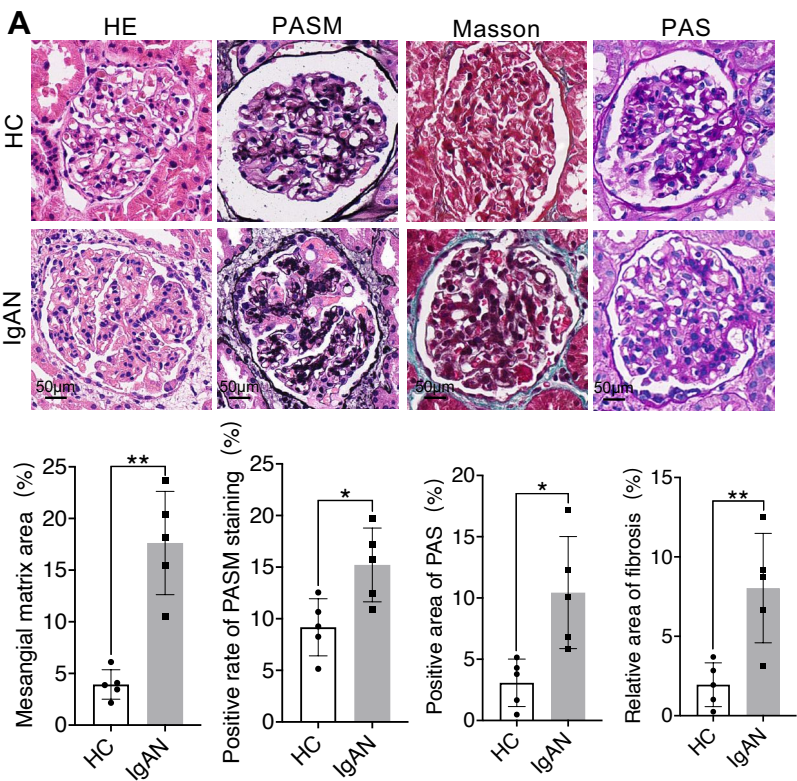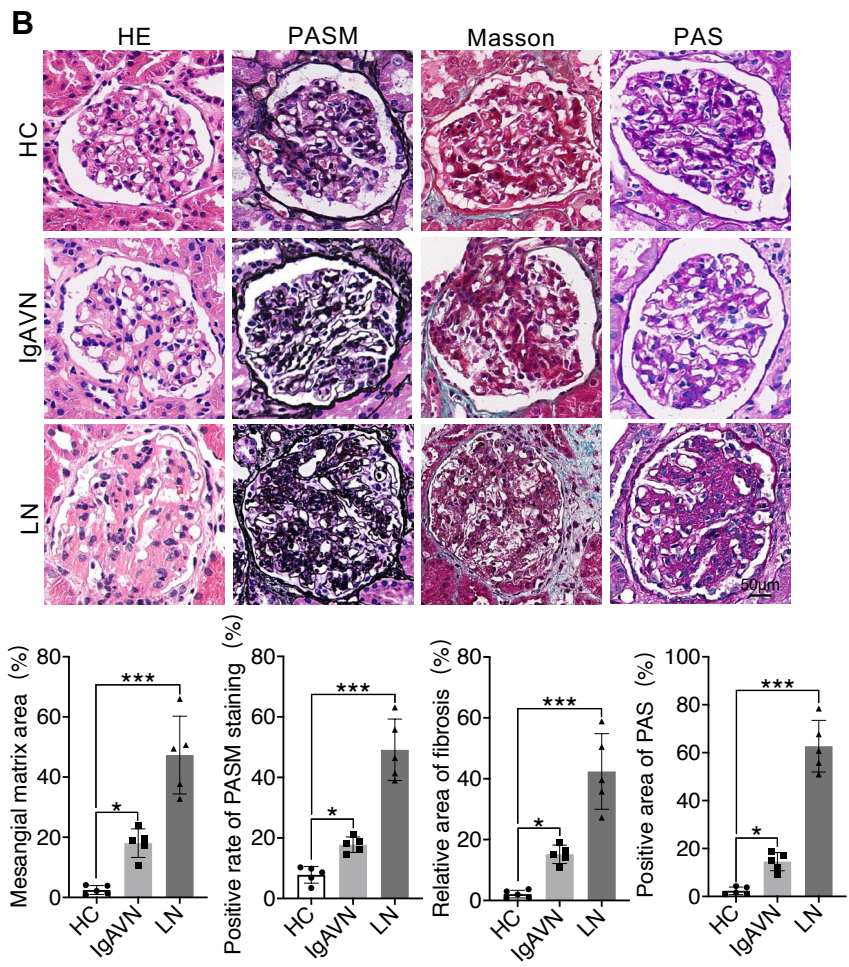

Supplement: sfae037_Supplemental_Files [file sfae037_supplemental_files.zip › Supplemental Figures.pdf]
